# A dataset of meta-analyses on crop diversification at the global scale

**DOI:** 10.1016/j.dib.2019.103898

**Published:** 2019-04-04

**Authors:** Damien Beillouin, Tamara Ben-Ari, David Makowski

**Affiliations:** a UMR Agronomie, INRA, AgroParisTech, Université Paris-Saclay, 78850 Thiverval-Grignon, France; b Centre International de Recherche sur l’Environnement et le Développement (CIRED)- CIRAD, UMR 8568 Nogent-sur-Marne, France

**Keywords:** Agroforestry, Cover crop, Intercropping, Rotation, Variety mixture, Systematic review, Meta-synthesis

## Abstract

Numerous meta-analyses have been conducted in the last three decades to assess the productive and environmental benefits resulting from a diversification of cropping systems. These meta-analyses assessed one or several diversification strategies (e.g., rotations, cover crops, agroforestry) according to various outcomes (e.g., productivity, profitability, biodiversity). To date, no dataset has provided a comprehensive synthesis of existing experimental data on crop diversification. We present here a dataset containing 2382 effect sizes published in 99 meta-analyses covering 3736 experimental studies worldwide (https://figshare.com/s/c15a93e96c95f89ddd89). We also provide an extensive appraisal of the quality of each meta-analysis and a quantification of the redundancy of primary studies between meta-analyses. Our database hence provides (i) a quantification of the impacts of a variety of diversification strategies on crop production, the environment and economic profitability at the global scale and, (ii) a quality and redundancy assessment that may be used as a reference for future studies.

**Table:** Specifications table

Subject area	*Agronomy, Ecology*
More specific subject area	*Biodiversity, greenhouse gas emissions, pest and disease, soil quality, soil carbon, water, yield*
Type of data	*Tables (n = 5): i) Definition_of_variables, ii) Literature_search, iii) Description meta-analyses, iv) Effect_sizes, v) Primary studies*
How data was acquired	*Systematic review of the literature: 6 databases were queried*
Data format	*Raw*
Experimental factors	*Agroforestry, associated plant species, cover crop, intercropping, rotation, cultivar mixture, landscape heterogeneity*
Experimental features	*Effect sizes of a variety of diversified treatments compared to less diversified controls*
Data source location	*114 countries over five continents*
Data accessibility	*Data are available with this article and online.*https://figshare.com/s/c15a93e96c95f89ddd89
Related research article	Philibert, A., Loyce, C., and Makowski, D. 2012. Assessment of the quality of meta-analysis in agronomy. *Agriculture, Ecosystems & Environment*, *148*, 72–82.

**Table:** 

**Value of the data** - The database allows to quantify and compare the impacts of various crop diversification strategies on the environment (e.g., soil carbon, biodiversity), agricultural production (e.g., crop yield, incidence of plant diseases) and economic profitability. - The database can be used to identify knowledge gaps, i.e. combinations of crop diversification strategies and outcomes with a low number of published meta-analyses. - The database includes an in-depth quality appraisal of 99 meta-analyses on crop diversification which can help weighting evidence in future scientific evidence assessments.

## 1 Data

**Fig. 1:**
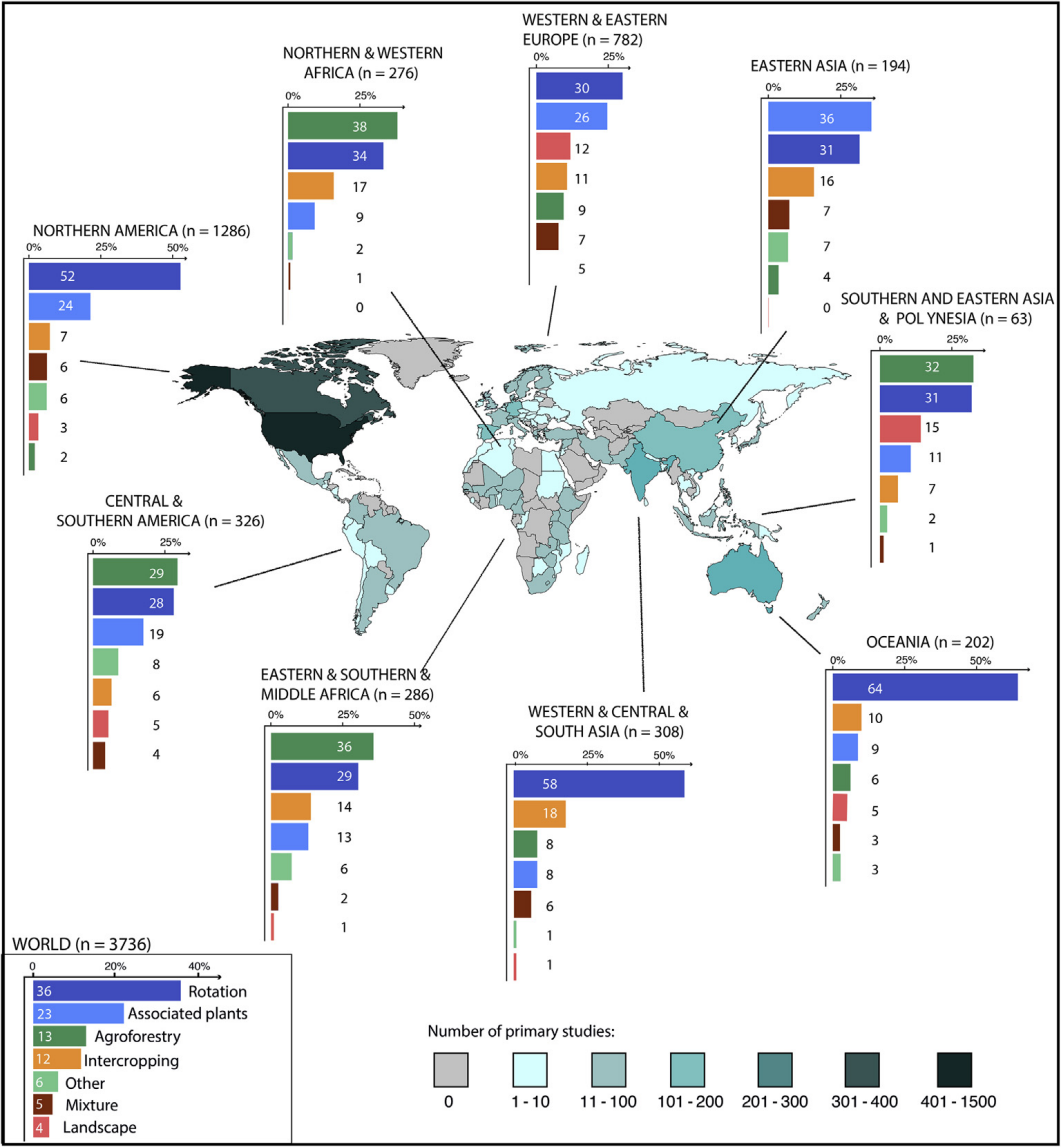
Locations of the experimental trials of all primary studies (map) and strategies of crop diversification by regions (bars). On the map, the numbers of primary studies are represented by a blue color scale. Countries with no trials on crop diversification in the dataset are colored in grey. On the bar plots, we detail the distribution of the strategies of crop diversification for nine regions and for the world. The legend of the color can be found in the insert at the bottom left of the graphic. The countries of the nine regions are listed below: **Central and Southern America:** Argentina, Bolivia, Brazil, Chile, Costa Rica, Colombia, Dominican Republic, Ecuador, El Salvador, Guatemala, Guyana, Honduras, Nicaragua, Mexico, Panama, Peru, Uruguay; **Eastern Asia:** China, Japan, Mongolia, South Korea; **Western and Eastern Europe:** Austria, Belgium, Croatia, Czech Republic, Denmark, Estonia, Finland, France, Germany, Greece, Hungary, Ireland, Italy, Luxembourg, Netherlands, Norway, Poland, Portugal, Romania, Russia, Serbia, Slovakia, Slovenia, Spain, Switzerland, Sweden, UK, Ukraine; **Middle and Southern Africa:** Eritrea, Ethiopia, Kenya, Madagascar, Malawi, Mozambique, Rwanda, Uganda, Tanzania, Zambia, Zimbabwe, Cameroon, Central African Republic, Republic of Congo, South Africa, Swaziland; **Northern and Western Africa:** Algeria, Egypt, Sudan, Western sahara Benin, Burkina Faso, Ivory Coast, Gambia, Ghana, Mali, Morocco, Niger, Nigeria, Senegal, Sierra Leone, Tunisia, Togo; **Northern America:** Canada, USA; **Oceania**: Australia, New Zealand; **South Estern Asia:** Indonesia, Malaysia, Myanmar, Nauru, New Caledonia, Papua New Guinea, Philippines, Samoa, Thailand, Vietnam, Timor-Leste; **Western, Southern and Central Asia:** Bangladesh, Cyprus, India, Iran, Israel, Jordan, Nepal, Pakistan, Sri Lanka, Syria, Turkey.

**Fig. 2:**
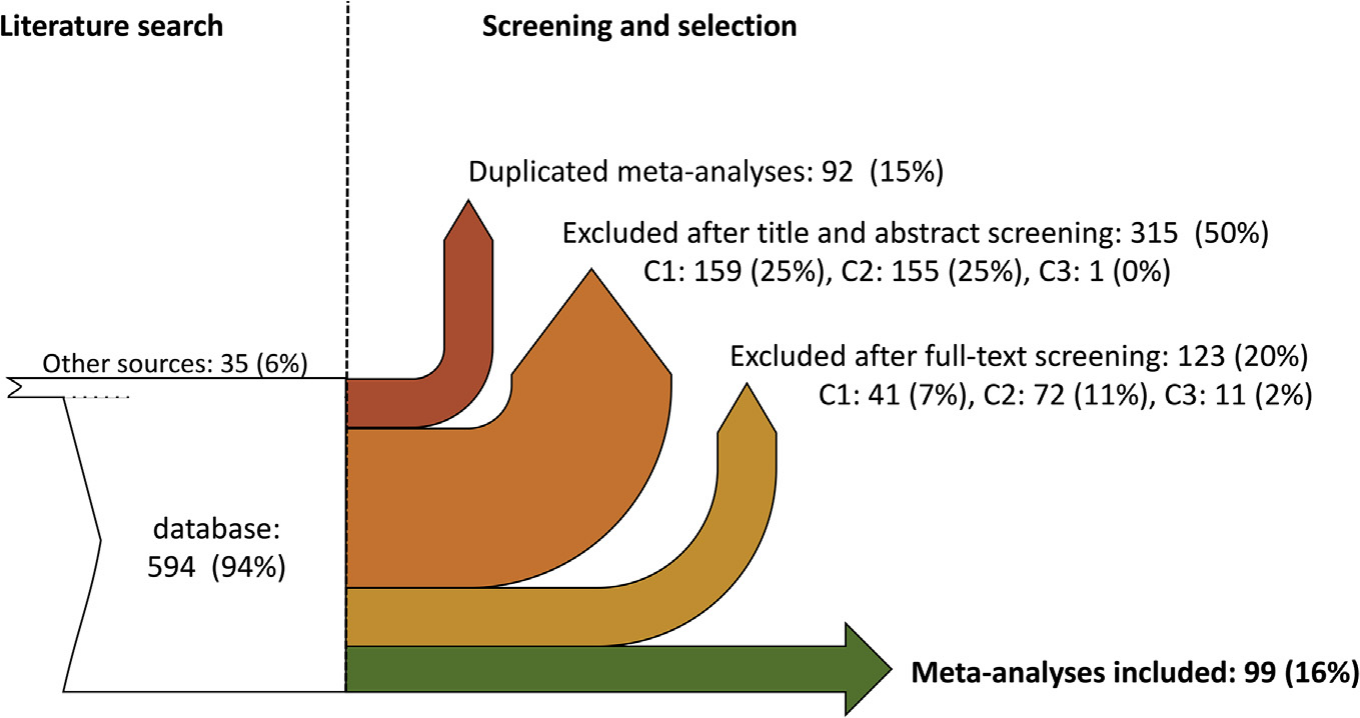
Flowchart of literature search and screening process. Articles initially identified are presented in white. After screening and selection, 99 meta-analyses are included in the database (green). The values indicate the number (and the proportion) of articles excluded/included at each step. C1: Selection criterion 1 (several individual studies are analyzed); C2: Selection criterion 2 (assessment of the impact of at least one strategy of crop diversification). C3: Selection criterion 3 (control plots are present next to treatment plots).

The dataset includes the values of effect sizes of 99 meta-analyses based on a total of 3736 unique primary studies. The dataset covers seven strategies of crop diversification (Table 1) and 114 countries over five continents (Fig. 1). More than 50 species are included in our dataset, but most of the data concerns six species (Maize, Wheat, Barley, Soybean, Bean, and Cowpea - Fig. 2). Our database also reports a quality assessment of the selected meta-analyses based on an extended and updated version of the quality checklist of [1].

**Table 1:** Definition of the seven strategies of crop diversification included in the database.

Strategies of crop diversification	Characteristics
Agroforestry	Agroforestry satisfies three conditions: i) at least two plant species interact biologically, ii) at least one of the plant species is a woody perennial, and iii) at least one of the plant species is managed for forage, annual or perennial crop production.
Associated plant species	Plant sown in addition to the main crop for agronomic or environmental purposes (*e.g.*, to manage soil erosion, soil fertility, soil quality, weeds, pests, diseases, biodiversity or nitrate leaching). The associated plant could be harvested or not, permanent or not. This category, primarily defined by plants function encompasses cover crops, trap crops, repellent crops, buffer and companion crops.
Cultivar mixture	The simultaneous cultivation in the same field of multiple cultivars of the same species. All cultivars are harvested.
Intercropping	The simultaneous cultivation in the same field of two or more crops (different species) for all or part of their growth cycle. All crop species are harvested.
Landscape heterogeneity	Landscape composition (perennial habitat diversity, semi-natural habitat cover) and configuration (mean patch size).
Other	Any other type of crop diversification.
Rotation	Recurrent succession of a set of selected crops grown on a particular agricultural land each season or each year according to a definite plan. Here, we do not consider as rotation, a system with temporal overlap of two or more crops.

The data collected are grouped into six separate but inter-related tables. The table ‘Effect_size’ contains the effect sizes reported in the 99 selected meta-analyses. Two other tables pertain to the extraction and classification of meta-information on ‘Effect_Size’; ‘Description_Meta-analyses’ compiles the references and the publication information on each meta-analysis, and the ‘Primary_Studies’ table reports information on each of the 4972 primary studies (of which 3736 are unique) included in the 99 meta-analyses. The table ‘Quality’ reports a comprehensive quality assessment for each of the 99 meta-analyses. Finally, the table ‘Definition_of_variable’ includes the definitions of all the attributes (column headers) of the other five tables. The following sections present each table in more details.

### 1.1 Table effect size

The “**Effect_Size**” table is the central table to quantify and compare the impacts of the seven types of strategies of crop diversification on the environment (*e.g.*, soil carbon, biodiversity), agricultural production (*e.g.*, crop yield, incidence of plant diseases) and economic profitability. This table can serve as a basis to perform a quantitative meta-synthesis (*i.e.,* the synthesis of several meta-analyses). This table can also be used to identify knowledge gaps, i.e. combinations of crop diversification strategies and outcomes with a low number of published meta-analyses.

Table 2 presents the number of effect sizes available for every crop diversification strategy and outcome; 34%, 29%, and 17% of the reported effect sizes measure the impacts of crop diversification on yield, soil quality and biodiversity respectively. The strategy ‘Associated plant species’ is the strategy including the highest number of effect size values (25% of all effect sizes), followed by ‘Intercropping’ (24% of all effect sizes), ‘Rotation’ (18% of all effect sizes), and ‘Agroforestry’ (13% of all effect sizes).

**Table 2:** Number of meta-analyses (Number of effect sizes) for different crop diversification strategies and different types of outcomes. The outcome sub-category showing the highest number of effect sizes in each column is highlighted in bold.

Main category of outcome	Sub-category of outcome	Associated plant species	Intercropping	Rotation	Agroforestry	Cultivar mixture	Other	Landscape heterogeneity	Two or more strategies intertwined	Total
Production	Yield	12 (63)	**14 (369)**	**15 (178)**	8 (90)	**4 (97)**	1 (5)		2 (15)	**50 (817)**
	Pests and Diseases	3 (34)	(1) 59	1 (1)	2 (15)	2 (23)	3 (9)	1 (17)		12 (158)
	Products quality		(2) 50	1 (2)						3 (52)
	Inputs use	2 (6)		1 (1)						3 (7)
	Production stability		1 (1)	1 (4)						2 (5)
Environment	Soil quality	**12 (281)**		10 (152)	**6 (169)**			1 (1)	**2 (76)**	26 (679)
	Biodiversity	4 (65)	1 (62)	2 (23)	4 (20)	3 (12)	**6 (105)**	5 (98)	2 (36)	23 (409)
	Greenhouse gas emission	3 (50)	1 (1)	7 (58)	(1) 17					10 (126)
	Water quality	7 (71)								7 (71)
	Miscellaneous	1 (8)			1 (2)		1 (1)			2 (11)
	Water use	2 (9)							1 (1)	3 (10)
Economic profitability			4 (23)	3 (9)	1 (5)					7 (37)

Each effect size is described by its type (*e.g.*, ratio, log ratio, difference, standardized difference), its value, and level of uncertainty (when available) *i.e.*, confidence intervals, standard-errors, number of data, etc.

**Fig. 3:**
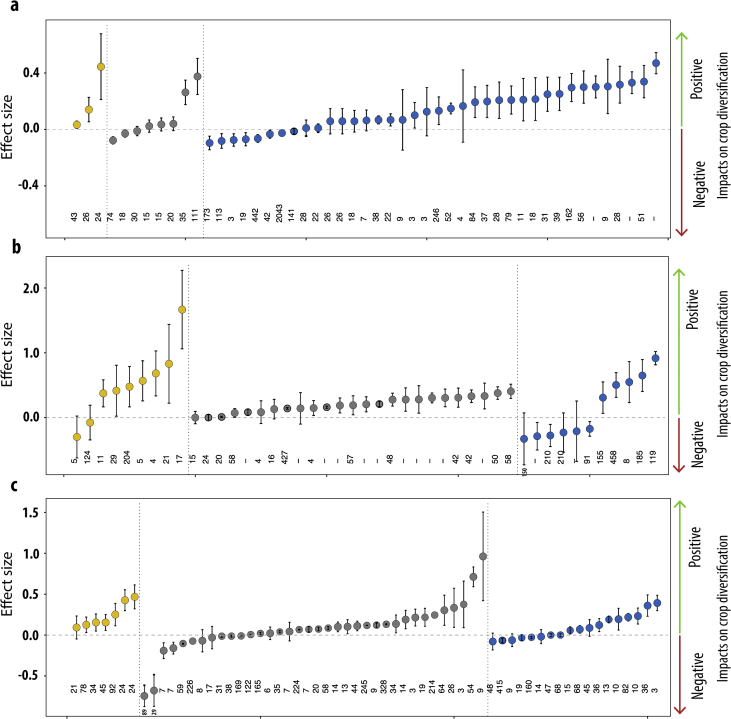
Compilation of effect sizes (ln(YT/YC), *i.e*., the log ratios of a measurement in a diversified treatment to its value in a less diversified control) for three diversification strategies: (a) rotation, (b) agroforestry, and (c) associated plant species. Each point corresponds to one effect size from one meta-analysis for one single category of outcome (note that several effect sizes may be affiliated to one single meta-analysis). The figure focuses on the following environmental and production outcomes: biodiversity (yellow), soil quality (grey) and productivity levels (blue). Vertical bars correspond to 95% confidence intervals. The number of data used to calculate each effect size are indicated at the bottom of each graph, when available. In some meta-analyses, the effect sizes were computed for a fraction of its total data sample (e.g., per covariate), but only global effect sizes are presented here. Note though that the totality of effect sizes is available in the table “Effect_size”. Effect sizes that were informed as relative distances were converted to log ratios and integrated in the figure whereas absolute differences and hedge's distances were not.

For illustration, Fig. 3 presents the values of the most common type of effect size, *i.e.* ln(Y_T_/Y_C_), and their associated 95% confidence intervals. These values measure the impacts of three crop diversification strategies (*i.e.*, rotation, agroforestry, associated plant species) on yield, soil quality (*e.g.*, soil carbon content, soil organic matter content, etc.), and biodiversity (*e.g.*, pollination, arthropod abundance, etc.).

### 1.2 Table literature search

**Fig. 4:**
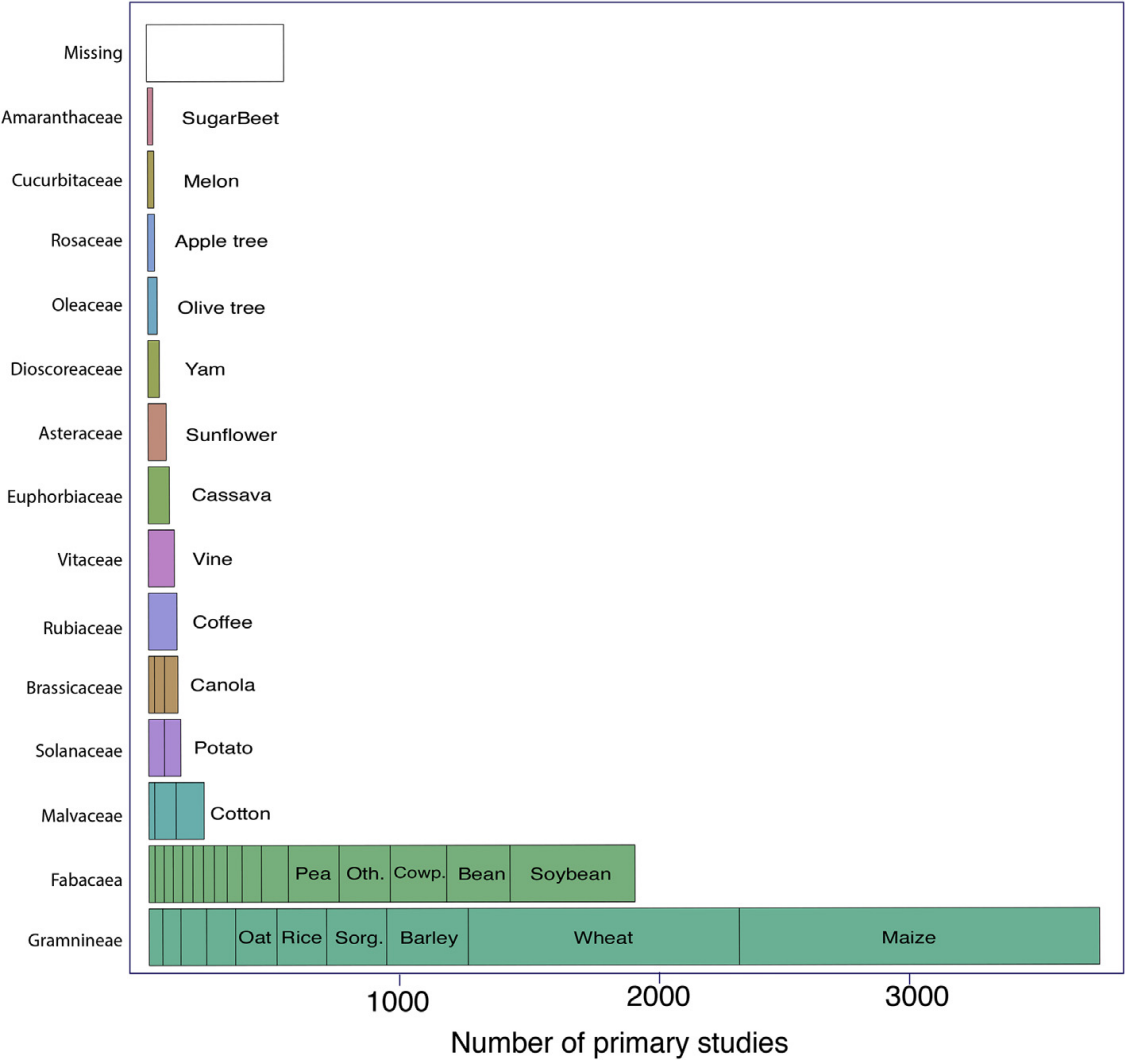
Fourteen most frequent botanical families in the primary studies included in the 99 meta-analyses on crop diversification. The most frequent species are indicated in the bar plots (restricted to one species when the total number of species is below 4; and species included in more than 150 primary studies for Gramineae and Fabacea). One primary study can report data for different species and/or families. **Gramineae**: Barley, Maize, Millet, Oat, Other, Rice, Rye, Ryegrass, Sorghum, Wheat; **Fabaceae**: Alfalfa, Bean, ChickPea, Clover, Cowpea, Faba Bean, Field Pea, Groundnut, Lentil, Lupin, Other, Garden Pea, Pigeon Pea, Soybean, Vetch; **Malvaceae**: Cocoa, Cotton; **Solanaceae**: Potato, Tomato; **Brassicaceae**: Cabbage, Mustard, Oilseed.

The ‘**Literature_Search’** table describes the references of all articles screened and the source where each article was identified (names of the database or additional sources). The table also specifies whether each article screened satisfied the considered inclusion criteria, and whether each article was selected or not. See Fig. 4 for a summary of the selection process.

### 1.3 Table Description_Meta-Analyses

**Fig. 5:**
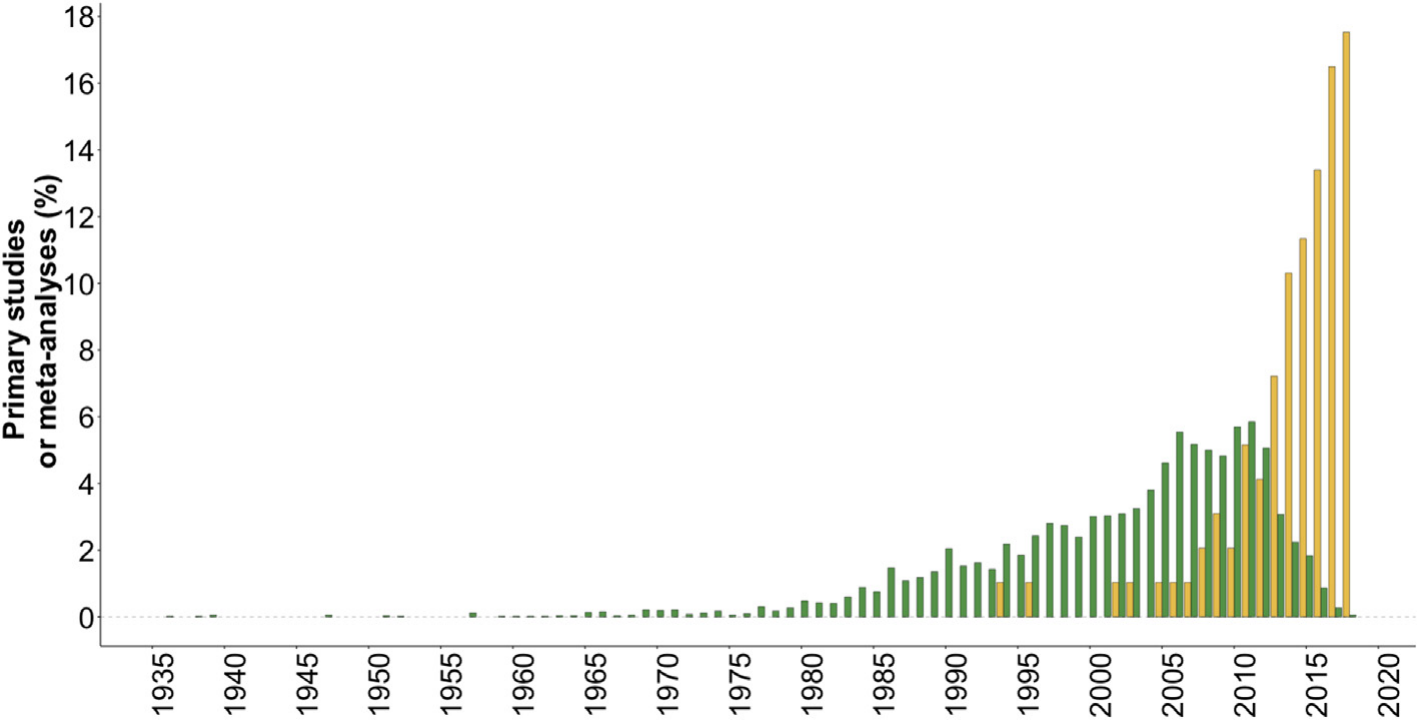
Dates of publication of the 99 selected meta-analyses (orange) and of their primary studies (green).

The ‘**Description_Meta-Analyses’** table describes the characteristics of each of the meta-analyses included in the Effect_Size table (*i.e.*, n = 99); full reference, type of publication, abstract, keywords, author's affiliations, crop species, crop diversification strategies. The 99 selected meta-analyses were published from 1994 to 2018 (Fig. 5). This table provides a rapid access to the scope and the objective of each selected meta-analysis identified by a unique index (attribute ‘ID’).

### 1.4 Table Primary_Studies

**Fig. 6:**
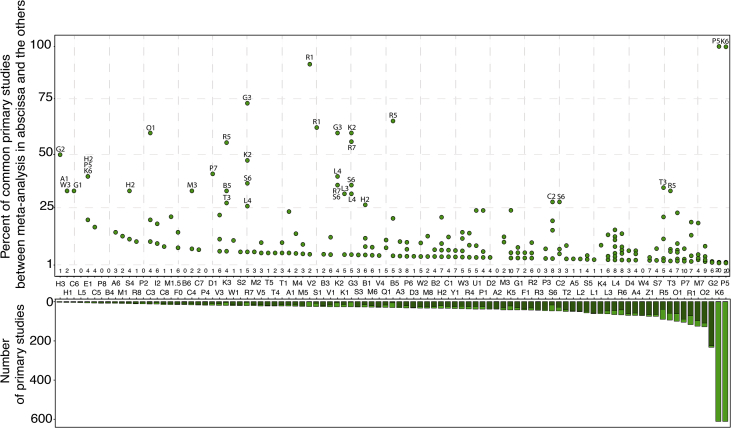
Percentage of common primary studies between meta-analyses (upper plot) and total number of primary studies used in each meta-analysis (bottom plot). Each point corresponds to a pair of meta-analyses. We calculated the percentages of common primary studies between the meta-analyses reported in the x-axis (ID of the meta-analyses) and the others (level of redundancy). We identified the name (ID) of all the meta-analyses with a redundancy level higher than 25%. The numbers at the bottom of the upper plot refer to the percentage of meta-analyses with at least one common primary studies with the meta-analyses reported in the x-axis. For the bottom plot, we distinguished unique primary studies (darkgreen) and primary studies used in at least two meta-analyses (lightgreen).

The “**Primary_Studies**” table can be used as a resource to identify relevant primary studies on crop diversification, to perform new meta-analyses or to update existing ones, or to analyze the redundancy of primary meta-analyses between meta-analyses. The table describes the main characteristics of primary studies (*i.e.*, the experimental trials) published on crop diversification: the references, plant species (Fig. 4), and locations of experimental trials of all primary studies included in each meta-analysis. All primary studies included in the 99 selected meta-analyses were published from 1936 to 2018 (Fig. 5). Most of the trials reported in primary studies were conducted in Northern America (1286 primary studies out of 3636), Western and Eastern Europe (782 primary studies), and in Central and Southern America (326 primary studies). A large majority of the primary studies focus on Gramineae and Fabaceae crops (Fig. 4). Fig. 6 presents the number of common primary studies between each pair of meta-analyses.

### 1.5 Table quality

**Fig. 7:**
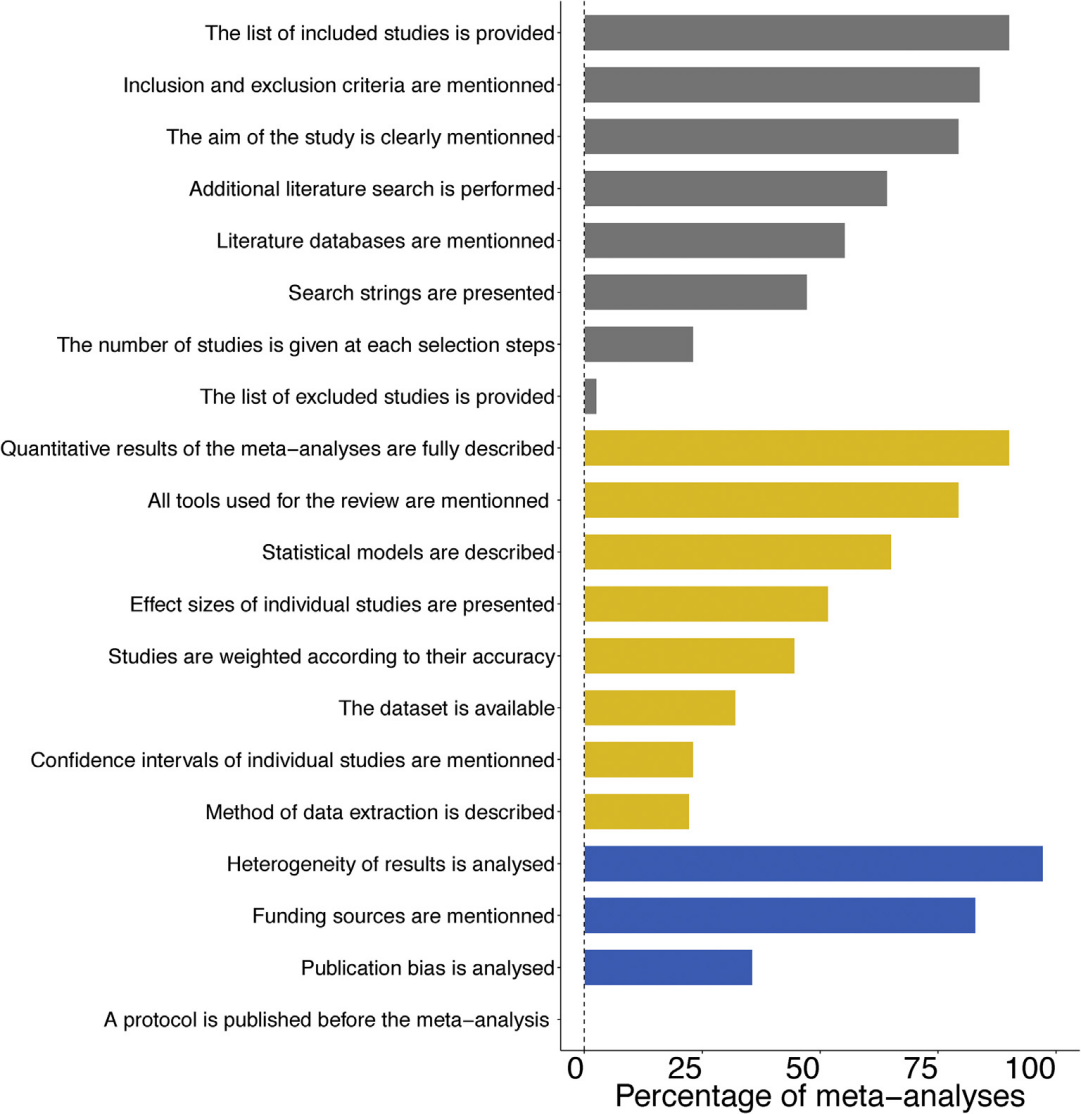
Percentage of meta-analyses satisfying each of the 20 quality criteria. Quality criteria are organized in three main groups: review and selection (grey bars), data and statistical analyses (yellow bars) and bias (blue bars).

The ‘**Quality**’ table can serve as a benchmark to improve the quality of systematic reviews and help for the development of appraisal tools for meta-analyses in a variety of research fields. The table describes a quality assessment of each of the 99 meta-analyses based on a set of 20 defined criteria along three main categories (*i.e.*, review and selection of the studies, data and statistical analysis, and identification of potential bias). Each criterion relies on the assessment of several quality items. When satisfied, a criterion is scored 1, and zero otherwise. Fig. 7 presents the percentage of meta-analyses satisfying each of the 20 quality criteria.

### 1.6 Table Definition_Of_Variable

The ‘**Definition_Of_Variable’** table describes the meaning of all attributes (column headers) of the five other tables. Definitions of terms and types of attributes (numerical, text, date) are detailed in this table (see Table 3 for a summary).

**Table 3:** Sizes of the six tables and types of attributes.

Table	# rows	Attribute types					
		Index	Class	Binary	Numeric	Integer	# column
Definition_Of_Variable	185	0	5	0	0	0	5
Literature_Search	538	0	3	9	0	0	12
Description_Meta-Analyses	100	1	14	4	1	0	20
Quality	100	1	50	11	1	3	67
Effect_Size	2383	1	33	8	34	0	76
Primary_Studies	4972	1	5	0	1	0	7

## 2 Experimental design, materials, and methods

### 2.1 Literature search

A systematic search of peer-reviewed journals and grey literature was carried out on May 2018 using six databases: Web of Science (http://apps.webofknowledge.com), CAB abstract (http://www.cabdirect.org), Greenfile (http://www.greeninfoonline.com), Environment Complete Database (https://www.ebsco.com), Agricola (http://agricola.nal.usda.gov), and Google Scholar (http://www.scholar.google.com). Our search equation was defined as follows; (meta-analysis OR meta analysis) AND (cropping system OR crop* OR agriculture) AND ((rotation OR Diversification OR intercrop* OR cover crop OR mixture) OR (organic AND (system OR agriculture)) OR (conservation AND (system OR agriculture)) OR no till* OR agroforestry OR agroecology). No restriction was applied to the date and language of publication in the article title, abstract and keywords, or to the geographical localization of the studies. References cited in each selected meta-analysis and those listed in a narrative review [2] were also screened. After the removal of duplicates, this initial literature search identified 537 candidate meta-analyses of potential interest evaluating the effect of crop diversification on a series of outcome (Fig. 2).

### 2.2 Meta-analyses selection

Each article title and abstract were screened for eligibility according to the following inclusion criteria: (i) article reporting a quantitative analysis of several primary experiments, (ii) article studying at least one crop diversification strategy, (iii) article including control plots adjoined to treatment plots (a less diversified cropping system should be tested as a control). Studies dealing with pure forestry or wood production were excluded. Two hundred twenty-two articles met these criteria (Fig. 2). Eligible full-texts articles were then examined according to the same three criteria and 123 articles were removed (41 because of a lack of quantitative result, 72 because of the lack of any defined crop diversification strategy, and 11 because of a lack of control plot) (Fig. 2). At the end of the screening process, 99 meta-analyses were selected. The selected meta-analyses reported data on seven diversification strategies; agroforestry (e.g. shaded coffee and cocoa production [3]), associated plant species (e.g. cover crops [4]), cultivar mixture (e.g. mixing two wheat cultivars in the same field [5]), intercropping (i.e. legume-cereal cultivation [6]), rotation (i.e. alternate legume and cereal cultivation [7]), landscape heterogeneity (i.e. presence of natural habitats [8]) and others (i.e. global plant genetic diversity [9]).

### 2.3 Extraction of data and quality assessment

All effect sizes related to crop diversification in each of the selected meta-analysis were extracted. Here, an effect size is defined as a quantitative measure of the effect of a crop diversification strategy compared to a reference cropping system (*i.e.*, less diversified) on one or several response variables (*e.g.*, crop yield, soil carbon content, biodiversity index, plant disease incidence). Let Y_T_ and Y_C_ be the values of one response variable in the diversified treatment and control, respectively. An effect size is a function of Y_T_ and Y_C_. Depending on the considered meta-analysis, the effect size can either be the ratio of Y_T_ to Y_C_ (or a log ratio, odds ratio) or the difference between Y_T_ and Y_C_ (standardized or not). Each selected meta-analysis presents the estimated values of one or several effect sizes for one or several groups of primary studies corresponding to different regions, crop types, etc. When several estimated values were available, they were extracted altogether with the characteristics of the groups of primary studies used for their estimation (*e.g.*, name of the region, type of crop, etc.). When available, information characterizing the uncertainty of estimated effect sizes were also systematically extracted (*e.g.*, sample size, confidence interval, p-value). Data were extracted from tables, text, supplementary information or directly from graphics using the WebPlotDigitizer software [10]. Information to assess the quality of each meta-analysis was also extracted using 20 criteria spanning the quality of the literature review, data extraction, data analyses, and interpretations.

Each meta-analysis was read carefully at least three times, to identify relevant data. In case of ambiguity, a second reader was asked to re-analyze the article. Inconsistencies of judgments were discussed by the two readers. When the reported data or protocols were unclear, authors were directly contacted and asked to provide additional information. The units of all data and the origin of the extracted effect sizes (figure, table, text section) were also precisely described. The qualitative and quantitative contents of all class, numerical, index, binary and date attributes were checked by importing each table in turn into the R software [11], and by visualizing data distribution for each attribute in turn. Outliers were systematically and manually checked in order to detect possible mistakes and returned to the original articles as many times as needed to check the accuracy of the data.
