# Supplementary material for: A dataset of meta-analyses on crop diversification at the global scale
Source: Data Brief. 2019 Apr 4;24:103898. doi: 10.1016/j.dib.2019.103898 (PMC6465772; doi:10.1016/j.dib.2019.103898)
Supplement: Multimedia component 1 [file mmc1.doc]

Conflict of Interest and Authorship Conformation Form

Please check the following as appropriate:

- All authors have participated in (a) conception and design, or analysis and interpretation of the data; (b) drafting the article or revising it critically for important intellectual content; and (c) approval of the final version.
- This manuscript has not been submitted to, nor is under review at, another journal or other publishing venue.
- The authors have no affiliation with any organization with a direct or indirect financial interest in the subject matter discussed in the manuscript
- The following authors have affiliations with organizations with direct or indirect financial interest in the subject matter discussed in the manuscript:

Author’s name Affiliation

Damien BEILLOUIN INRA-UMR Agronomie

David MAKOWSKI INRA-UMR Agronomie- CIRED

Tamara BEN-ARI INRA-UMR Agronomie-CIRED
